# Predictors of chronic kidney disease survival in type 2 diabetes: a 12-year retrospective cohort study utilizing estimated glomerular filtration rate

**DOI:** 10.1038/s41598-024-58574-x

**Published:** 2024-04-19

**Authors:** Ammar Abdulrahman Jairoun, Chong Chee Ping, Baharudin Ibrahim

**Affiliations:** 1 Discipline of Clinical Pharmacy, School of Pharmaceutical Sciences, Universiti Sains Malaysia (USM), 11800 Penang, Minden, Malaysia; 2 Faculty of Pharmacy, Universiti Malaya, Kuala Lumpur, Malaysia

**Keywords:** Chronic kidney disease, eGFR, Diabetes mellitus, Survival analysis, Cardiovascular disease, Endocrine system and metabolic diseases, Kidney, Kidney diseases

## Abstract

Predicting the course of kidney disease in individuals with both type 1 and type 2 diabetes mellitus (DM) is a significant clinical and policy challenge. In several regions, DM is now the leading cause of end-stage renal disease. The aim of this study to identify both modifiable and non-modifiable risk factors, along with clinical markers and coexisting conditions, that increase the likelihood of stage 3–5 chronic kidney disease (CKD) development in individuals with type 2 DM in the United Arab Emirates (UAE). This was a single-center retrospective cohort study based on data derived from electronic medical records of UAE patients with DM who were registered at outpatient clinics at Tawam Hospital in Al Ain, UAE, between January 2011 and December 2021. Type 2 DM patients aged ≥ 18 years who had serum HbA1c levels ≥ 6.5% were included in the study. Patients with type 1 DM, who had undergone permanent renal replacement therapy, who had under 1 year of follow-up, or who had missing or incomplete data were excluded from the study. Factors associated with diabetic patients developing stage 3–5 CKD were identified through Cox regression analysis and a fine and gray competing risk model to account for competing events that could potentially hinder the development of CKD. A total of 1003 patients were recruited for the study. The mean age of the study cohort at baseline was 70.6 ± 28.2 years. Several factors were found to increase the risk of developing stage 3–5 CKD: advancing age (HR 1.005, 95% CI 1.002–1.009, *p* = 0.026), a history of hypertension (HR 1.69, 95% CI 1.032–2.8, *p* = 0.037), a history of heart disease (HR 1.49, 95% CI 1.16–1.92, *p* = 0.002), elevated levels of serum creatinine (HR 1.006, 95% CI 1.002–1.010, *p* = 0.003), decreased levels of estimated glomerular filtration rate (eGFR) (HR 0.943, 95% CI, 0.938–0.947; *p* < 0.001), and the use of beta-blockers (HR 139, 95% CI 112–173, *p* = 0.003). Implementing preventative measures, initiating early interventions, and developing personalized care plans tailored to address specific risk factors are imperative for reducing the impact of CKD. Additionally, the unforeseen findings related to eGFR highlight the ongoing need for research to deepen our understanding of the complexities of kidney disease.

## Introduction

Diabetes mellitus (DM) is escalating into a significant public health concern^1^ due to its surging prevalence worldwide. Between 2000 and 2015, prevalence rates climbed from 4.6 to 8.8%, with projections indicating a rise to 10.4% by 2040^2^. Type 2 DM, which accounts for the majority of DM cases globally, affects 1 in 11 people, with its increase largely attributed to lifestyle factors^3^. This trend is consistent across various regions, including Arab countries in the Middle East and North Africa^4^.

Kidney damage, a serious complication of DM, is a primary cause of chronic kidney disease (CKD) globally ^5–8^. CKD, which affects 5–7% of the global population, is primarily driven by DM and hypertension^9^. Compared to individuals without DM, those with the condition have a nearly twofold increased risk of developing CKD^10^, with 40% of those with type 2 DM experiencing diabetic nephropathy, a severe microvascular complication^11^.

The decline in estimated glomerular filtration rate (eGFR) and the onset of albuminuria are key clinical indicators of kidney disease. An eGFR of less than 60 mL/min per 1.73 m^2^ body surface area (BSA) on two occasions, 3 months apart, is a crucial diagnostic criterion for CKD^12^.

Diabetic kidney disease (DKD) is characterized by a complex interplay of various factors leading to structural and functional changes in the kidneys. Chronic hyperglycemia in individuals with DM is a significant risk factor for kidney disease, as it progressively damages the tubules and glomeruli, potentially leading to severe health outcomes. Approximately half of those with type 2 DM are affected by renal issues marked by increased urine albumin excretion, diminished renal function, or both^13^.

DM patients face numerous risk factors for CKD, including high blood pressure, long-standing DM, hypertriglyceridemia, elevated uric acid levels, and persistent inflammation^14–16^. Socioeconomic and ethnic factors also contribute to higher CKD rates in certain groups^17–21^.

The risk of complications escalates significantly as CKD progresses^22^. Furthermore, individuals with type 2 DM are at an increased risk of death from CKD and cardiovascular events^23^. Early diagnosis of CKD is essential for diabetic patients to monitor cardiovascular risk factors and initiate treatments to slow the progression of kidney failure^24^.

With DM being the leading cause of end-stage renal disease (ESRD) in some areas, understanding the progression of kidney disease in people with both type 1 and type 2 DM presents a significant clinical and policy challenge^6,25^. CKD may affect 25–50% of individuals with DM^26–28^, contributing to higher mortality and healthcare costs associated with DKD^29–32^.

Pharmacological treatments and lifestyle modifications can aid in the prevention and management of CKD among diabetic patients. Collaborative efforts, such as those by the American Diabetes Association (ADA) and KDIGO, have produced evidence-based guidelines to improve clinical outcomes for individuals with DM and CKD^13,33^.

Despite several studies on the prevalence of and risk factors for CKD among diabetic patients in industrialized nations, there is a notable lack of research on these topics in the United Arab Emirates (UAE). Information on the prevalence of CKD among Middle Eastern diabetic populations is also scarce. Therefore, this study aimed to identify the clinical signs, comorbid conditions, and modifiable and non-modifiable risk factors that increase the likelihood of individuals with type 2 DM in the UAE developing stage 3–5 CKD. Understanding these factors is crucial for devising preventative strategies and enhancing management practices.

## Materials and methods

### Research design

This was a single-center retrospective cohort study based on data obtained from electronic medical records (EMR) of UAE patients with DM who were registered at outpatient clinics at Tawam Hospital in Al Ain, UAE, between January 2011 and December 2021.

### Study area

Al Ain, with a population of approximately 1.4 million, ranks as the fourth-largest city in the UAE^34^. Among its prominent institutions, Tawam Hospital stands out as a leading tertiary care center, renowned for providing specialized medical services not only to the local community but also to patients from further afield. Established in 1979, Tawam Hospital has become a key component of the Abu Dhabi Health Services Company (SEHA) Health System, reflecting its important role in the healthcare landscape of the region^35^. As a doctor-led facility licensed by the Department of Health in Abu Dhabi, Tawam Hospital boasts a team of board-certified physicians from North America and Europe, all of whom have received training in Western medical practices. This international standard of healthcare is further enhanced by the hospital’s association with Johns Hopkins Medicine International, which brings clinical, administrative, and educational expertise to elevate patient care quality and safety. With 509 beds, the hospital offers a wide array of services and specialties, including, but not limited to, pain management, physical therapy, in vitro fertilization (IVF), and both adult and pediatric oncology, alongside dedicated centers for obesity care and for sleep. This extensive range of services underlines Tawam Hospital’s commitment to addressing diverse healthcare needs. Tawam Hospital’s vision to be a leading healthcare provider both regionally and globally is underpinned by its dedication to excellence, innovation, and community engagement. Through its comprehensive services, esteemed staff, and strategic partnerships, Tawam Hospital is poised to continue its legacy of delivering superior healthcare outcomes and contributing to the well-being of the Al Ain community and beyond.

### Study population (inclusion and exclusion criteria)

The study population included type 2 DM patients registered at outpatient clinics at Tawam Hospital in Al Ain, UAE. The inclusion criteria were type 2 DM patients aged ≥ 18 years who had a serum HbA1c level ≥ 6.5%, had a diagnosis established by a physician, or were receiving medications for DM (e.g., sulfonylurea, thiazolidinedione, dipeptidyl peptidase-4 inhibitor, biguanide, or insulin). Patients with CKD who had undergone permanent renal replacement therapy (hemodialysis, peritoneal dialysis, or kidney transplant); patients with type 1 DM; patients with under 1 year of follow-up or missing or incomplete data; and patients with COVID-19, cancer, HIV, or AIDS were excluded from the study.

### Sample size estimation

We determined the sample size for our cohort study using OpenEpi software and the following cohort study sample size formula: (https://www.openepi.com/SampleSize/SSCohort.htm). The sample size was determined in line with the study’s primary objective—to identify the incidence of stage 3–5 CKD development in individuals with type 2 DM. We employed a two-sided test with a power of (1 − β) = 0.95 and a significance level of α = 0.05. The control-to-case ratio was set at 1. Based on an anticipated risk ratio of 3.77^36^ and an assumed proportion of controls with outcome = 3.3, our calculations indicated a minimum required sample size of 314 participants.

### Sampling method and participant recruitment

Convenience sampling was used to recruit patients. The patients were recruited retrospectively from EMR of UAE patients with type 2 DM who were registered at outpatient clinics at Tawam Hospital in Al Ain, UAE.

The annual follow-up data for this retrospective cohort study were collected from January 2011 to December 2021 to establish the risk factors for CKD in type 2 DM patients undergoing surveillance for the condition.

Baseline clinical variables, demographic data, and time to event (development of stage 3–5 CKD) were collected from an electronic database. We identified patients using unique reference numbers, which helped ensure that patients met the inclusion criteria, which, in turn, reduced the reporting error. The eGFR was repeatedly evaluated for each patient every 3 months, from baseline to December 2021. Additionally, follow-up laboratory tests were revised and monitored to ensure that patients fulfilled the inclusion criteria; this enhanced the quality of the collected data. A data collection form (in an Excel sheet) was designed for data collection purposes. The data collected for each patient included participant ID number and baseline covariates. Patients’ characteristics were recorded at baseline. The collected data included: (1) sociodemographics, (2) detailed medical history, (3) anthropometric measurements, (4) laboratory analyses and clinical parameters, and (5) disease characteristics and medications. For detailed operational definitions of patients’ characteristics, see supplementary materials.

### Data quality

To ensure the integrity and quality of our data throughout this study, meticulous protocols were established. Initially, we set a follow-up duration of 11.7 years to capture a broad spectrum of CKD occurrences. We then defined precise inclusion criteria to encompass all qualified patients while minimizing the risk of excluding potential CKD cases. To further refine our cohort, we excluded individuals with less than 1 year of follow-up or who lacked baseline data, employing stringent selection standards to ensure access to complete and up-to-date medical records. This approach ensured the collection of comprehensive participant information. Routine patient monitoring facilitated the timely documentation of CKD events, effectively reducing the chances of administrative censoring and loss of follow-up. From the outset of the study until December 2021, we conducted quarterly assessments of each patient’s eGFR, allowing for continuous surveillance of their condition. Additionally, we conducted in-depth analyses of patient records to identify and document any competing events that might have affected CKD progression, ensuring these were appropriately considered in our analysis. Lastly, to properly address outcomes such as cardiovascular complications, mortality, initiation of renal replacement therapy, and discontinuation of statin use, we applied a Fine and Gray competing risk model. These systematic and thorough methodologies not only enhanced the robustness and credibility of our retrospective cohort study but also assured the generation of accurate, high-quality data and precise estimates of CKD incidence.

### Statistical analysis

For data entry, coding, and analysis, we used SPSS Version 26. Various procedures, including independent samples *t*-tests, one-way ANOVA, the Mann–Whitney *U* test, the Kruskal–Wallis test, and χ^2^ or Fisher’s exact tests for categorical data, were used to evaluate the baseline characteristics of the patients. The Shapiro–Wilk test or a visual inspection of a normal QQ plot was used to verify normality. Data missing from the dependent variable and covariate were not imputed. The observation duration, which was calculated from baseline to the last outpatient appointment or the diagnosis of stage 3–5 CKD, was expressed as years patient was at risk of developing stage 3–5 CKD. Factors linked to the development of stage 3–5 CKD in diabetic patients were determined by Cox regression analysis. A Cox–Snell residual graph, a log–log plot, and a Schoenfeld residuals global test were used to evaluate the suitability of the model. Predictor variables were analyzed using univariate Cox regression, and those with *p* < 0.1 were included in multivariable analysis through a stepwise backward selection process^36^. A Fine and Gray competing risk regression model assessed the connection between possible risk variables and stage 3–5 CKD^37^. This model considered competing events such as concomitant diseases, comorbidities, and stopping statin medication. In order to handle multicollinearity, variables in the Fine and Gray model were chosen using LASSO with BIC. For the most predictive covariates, the shrinkage parameter that was chosen minimized the BIC^38,39^. Statistical significance was established when *p* < 0.05 using R software version 3.6.3.

### Ethical considerations

This study was reviewed and approved by the institutional ethical committee of Tawam Hospital in Al Ain, UAE (Ref. No.: AA/AJ/771), and by the Human Research Ethics Committee (JePEM) of Universiti Sains Malaysia. All methods were conducted in accordance with relevant guidelines and regulations. All forms were anonymous and were entered into SPSS software. Only research team members accessed the data. Data presented were grouped and did not identify the respondents individually.

## Results

### Patient enrollment process

**Figure 1:**
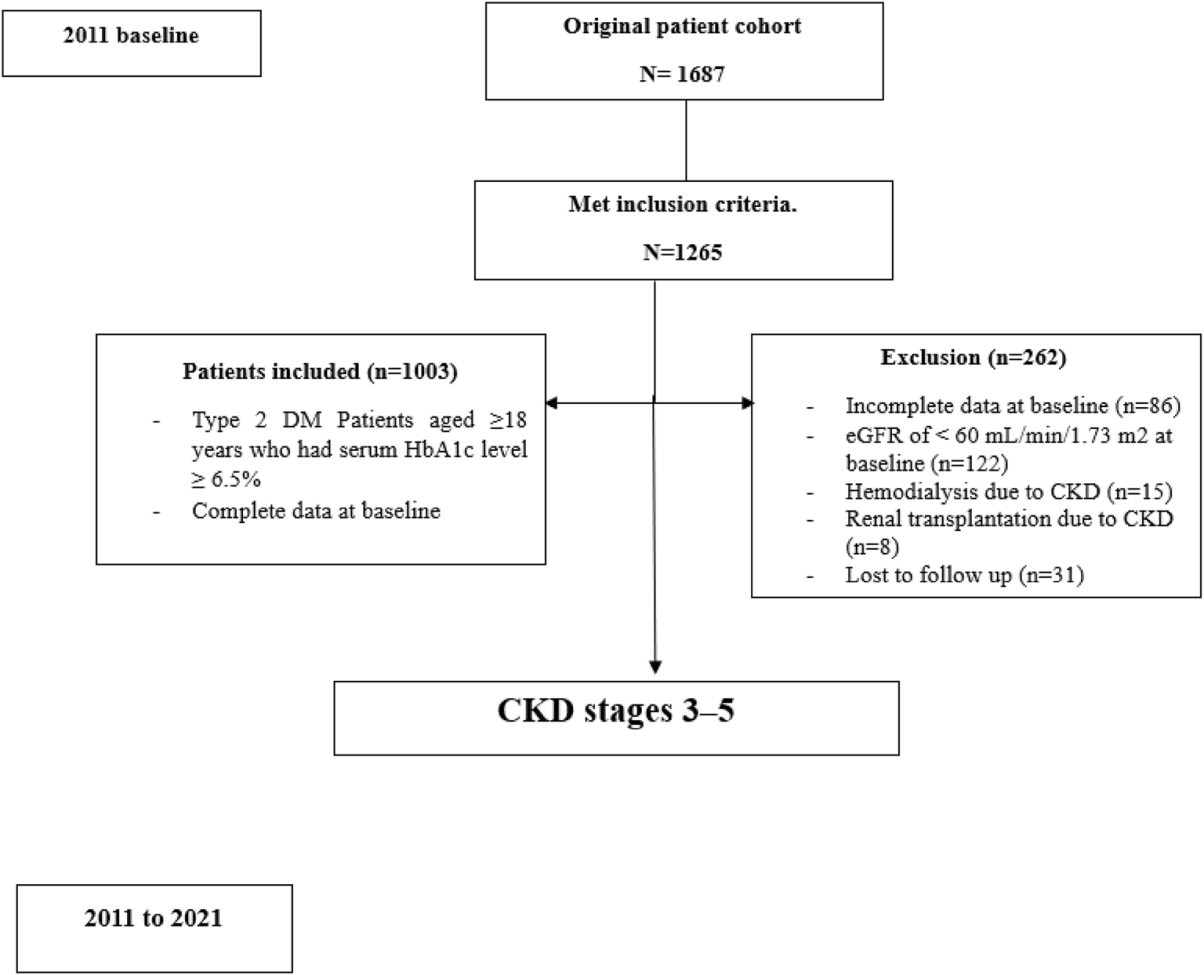
Flow diagram of the patient cohort.

Out of the 1265 patients who met the inclusion criteria, 262 were found to be ineligible for various reasons. Among them, 122 had eGFR levels below 60 mL/min/1.73 m^2^; 15 required hemodialysis due to CKD; 8 had undergone transplantation; and 86 had incomplete data on baseline HbA1c, urea, albumin, total cholesterol to high-density lipoprotein cholesterol (TC/HDL-C) ratio, or vitamin D. From the baseline until December 26th, 2021, we monitored the eGFR of each patient every 3 months. Unfortunately, during this period we could not obtain follow-up measurements for urea or serum creatinine (SCr) from 31 patients, which led to their exclusion due to loss of follow-up data. As a result of these considerations, a total of 1003 patients with an eGFR greater than or equal to 60 mL/min/1.73 m^2^ were ultimately included in this study (Fig. 1).

### Demographics and baseline characteristics

Table 1 presents the demographic characteristics and comorbidities of the study patients. The mean age of the study cohort at baseline was 70.6 ± 28.2 years. Of all patients, 60% (*n* = 602) were female and 40% (*n* = 401) were male. Of the total participants, 8.2% (*n* = 82) were smokers and 36.9% (*n* = 370) had a family history of DM. The majority of the study cohort had a history of hypertension (848/1003; 84.5%), 90.6% (*n* = 909) had a history of dyslipidemia, and 14.4% (*n* = 144) had a history of ischemic heart disease.

**Table 1:** Demographics and comorbidities of the study cohort (*N* = 1003).

	Group	Descriptive statistics
Age (years), mean (SD)	70.6 (28.2)	
Gender, *n* (%)	Female	602 (60)
	Male	401 (40)
Smoking, *n* (%)	Yes	82 (8.2)
	No	921 (91.8)
Family history of DM, *n* (%)	Yes	370 (36.9)
	No	633 (63.1)
Hypertension, *n* (%)	Yes	848 (84.5)
	No	155 (15.5)
Dyslipidemia, *n* (%)	Yes	909 (90.6)
	No	94 (9.4)
Ischemic heart disease, *n* (%)	Yes	144 (14.4)
	No	859 (85.6)

*DM* diabetes mellitus.

Patients with a stage 3–5 CKD event were older at baseline, less frequently had a family history of DM, and less frequently had a history of hypertension but more frequently had a history of ischemic heart disease (Table 2).

**Table 2:** Comparison of demographics and comorbidities of patients with and without stage 3–5 CKD.

	Total (*N* = 1003)	CKD (*n* = 388)	No CKD (*n* = 615)	*p* value*
Age (years), mean (SD)	70.6 (28.2)	76.5 (11.1)	66.8 (34.4)	< 0.001^a^
Female gender, *n* (%)	602 (60)	222 (36.9)	380 (63.1)	0.150
Smoking, *n* (%)	82 (8.2)	30 (36.6)	52 (63.4)	0.684
Family history of DM, *n* (%)	370 (36.9)	116 (31.4)	254 (68.6)	< 0.001
Hypertension, *n* (%)	848 (84.5)	371 (43.8)	477 (56.3)	< 0.001
Dyslipidemia, *n* (%)	909 (90.6)	356 (39.2)	553 (60.8)	0.332
Ischemic heart disease, *n* (%)	144 (14.4)	94 (65.3)	50 (34.7)	< 0.001

*DM* diabetes mellitus, *CKD* chronic kidney disease.

CKD: eGFR < 60 mL/min/1.73 m^2^ for ≥ 3 months.

No CKD: eGFR > 60 mL/min/1.73 m^2^ for ≥ 3 months.

*p values obtained from chi-square tests (two-tailed) for categorical variables.

^a^independent samples *t*-tests for continuous variables.

Table 3 presents concurrent medications of the study cohort. Of the total patients, 81 (8.1%) were non-statin users, 710 (70.8%) were low to moderate intensity statin users, and 212 (21.1%) were high intensity statin users.

**Table 3:** Concurrent medications of the study cohort (*N* = 1003).

Medications	Groups	Frequency	Percentage
Statins	No statin	81	8.1
	Low/moderate intensity statin	710	70.8
	High intensity statin	212	21.1
Diuretics	Yes	332	33.1
	No	671	66.9
ACEIs	Yes	263	26.2
	No	740	73.8
ARBs	Yes	509	50.7
	No	494	49.3
Alpha-blockers	Yes	83	8.3
	No	920	91.7
Beta-blockers	Yes	300	29.9
	No	703	70.1
Calcium channel blockers	Yes	301	30.0
	No	702	70.0
Sulfonylureas	Yes	545	54.3
	No	458	45.7
Thiazolidinedione	Yes	164	16.4
	No	839	83.6
Dipeptidyl peptidase	Yes	422	42.1
	No	581	57.9
Biguanide	Yes	950	94.7
	No	53	5.3
Alpha glucosidase	Yes	39	3.9
	No	964	96.1
Insulin	Yes	227	22.6
	No	776	77.4

*ACEIs* angiotensin-converting enzyme inhibitors, *ARBs* angiotensin receptor blockers.

Antihyperglycemic cardiovascular medication use among the study cohort was as follows: 332 (33.1%) used diuretics, 263 (26.2%) used angiotensin-converting enzyme inhibitors (ACEIs), 509 (50.7%) used angiotensin receptor blockers (ARBs), 83 (8.3%) used alpha-blockers, 300 (29.9%) used beta-blockers, 301 (30%) used calcium channel blockers, 545 (54.3%) used sulfonylureas, 164 (16.4%) used thiazolidinedione, 422 (42.1%) used dipeptidyl peptidase, 950 (94.7%) used biguanide, 39 (3.9%) used alpha glucosidase, and 227 (22.6%) used insulin.

Table 4 illustrates the patients’ medication usage based on the development of stage 3–5 CKD. Patients with stage 3–5 CKD were more frequent users of diuretics, beta-blockers, calcium channel blockers, and insulin but less frequently used ARBs and biguanides compared to patients without CKD.

**Table 4:** Comparison of medications of patients with and without stage 3–5 CKD.

	Total (*N* = 1003)	CKD (*n* = 388)	No CKD (*n* = 615)	*p* value*
Statin, *n* (%)				
No statin	81 (8.1)	25 (30.9)	56 (69.1)	0.077
Low/moderate intensity statin	710 (70.8)	269 (37.9)	441 (62.1)	
High intensity statin	212 (21.1)	94 (44.3)	118 (55.7)	
Diuretics, *n* (%)				
Yes	332 (33.1)	170 (51.2)	162 (48.8)	< 0.001
No	671 (66.9)	218 (32.5)	453 (67.5)	
ACEIs, *n* (%)				
Yes	263 (26.2)	113 (43)	150 (57)	0.097
No	740 (73.8)	275 (37.2)	465 (62.8)	
ARBs, *n* (%)				
Yes	509 (50.7)	225 (44.2)	284 (55.8)	< 0.001
No	494 (49.3)	163 (33)	331 (67)	
Alpha-blockers, *n* (%)				
Yes	83 (8.3)	36 (43.4)	47 (56.6)	0.360
No	920 (91.7)	352 (38.3)	568 (61.7)	
Beta-blockers, *n* (%)				
Yes	300 (29.9)	177 (59)	123 (41)	< 0.001
No	703 (70.1)	211 (30)	492 (70)	
Calcium channel blockers, *n* (%)				
Yes	301 (30)	154 (51.2)	147 (48.8)	< 0.001
No	702 (70)	234 (33.3)	468 (66.7)	
Sulfonylureas, *n* (%)				
Yes	545 (54.3)	223 (40.9)	322 (59.1)	0.113
No	458 (45.7)	165 (36)	293 (64)	
Thiazolidinedione, *n* (%)				
Yes	164 (16.4)	74 (45.1)	90 (54.9)	0.064
No	839 (83.6)	314 (37.4)	525 (62.6)	
Dipeptidyl peptidase, *n* (%)				
Yes	422 (42.1)	167 (39.6)	255 (60.4)	0.622
No	581 (57.9)	221 (38)	360 (62)	
Biguanide, *n* (%)				
Yes	950 (94.7)	360 (37.9)	590 (62.1)	0.030
No	53 (5.3)	28 (52.8)	25 (47.2)	
Alpha glucosidase, *n* (%)				
Yes	39 (3.9)	18 (46.2)	21 (53.8)	0.329
No	964 (96.1)	370 (38.4)	594 (61.6)	
Insulin, *n* (%)				
Yes	227 (22.6)	123 (54.2)	104 (45.8)	< 0.001
No	776 (77.4)	265 (34.1)	511 (65.9)	

*ACEIs* angiotensin-converting enzyme inhibitors, *ARBs* angiotensin receptor blockers, *CKD* chronic kidney disease.

CKD: eGFR < 60 mL/min/1.73 m^2^ for ≥ 3 months.

No CKD: eGFR > 60 mL/min/1.73 m^2^ for ≥ 3 months.

*p values obtained from chi-square tests (two-tailed) for categorical variables.

Table 5 presents the baseline clinical parameters according to the progression of stage 3–5 CKD. The mean baseline eGFR was 89.9 ± 13.3 mL/min/1.73 m^2^. Patients with a stage 3–5 CKD event had higher systolic blood pressure (SBP), HbA1c, SCr, urea, and potassium but lower diastolic blood pressure (DBP), eGFR, alanine transaminase (ALT), and albumin than patients without stage 3–5 CKD events.

**Table 5:** Comparison of baseline clinical characteristics of patients with and without stage 3–5 CKD.

	Total (*N* = 1003)	CKD (*n* = 388)	No CKD (*n* = 615)	*p* value*
Anthropometric values				
BMI (kg/m^2^), mean (SD)	29.8 (6.8)	29.4 (7.4)	30.2 (6.6)	0.091
SBP (mmHg), mean (SD)	135.4 (38.3)	140.6 (57.4)	132.1 (0.7)	*0.001*
DBP (mmHg), mean (SD)	75.8 (11.7)	74.1 (12.2)	76.9 (11.2)	*< 0.001*
Laboratory values				
HbA1c (%), mean (SD)	8.3 (2.1)	8.6 (2)	8.1 (2.1)	*< 0.001*
TC (mmol/L), mean (SD)	4.8 (1.8)	4.7 (2.4)	4.8 (1.3)	0.479
TG (mmol/L), mean (SD)	1.64 (1.6)	1.67 (1.8)	1.63 (1.5)	0.681
LDL-C (mmol/L), mean (SD)	3 (2)	2.98 (2.7)	3 (1.3)	0.808
HDL-C (mmol/L), mean (SD)	1.09 (0.40)	1.07 (0.45)	1.10 (0.36)	0.149
TC/HDL-C ratio, mean (SD)	4.59 (1.6)	4.64 (1.7)	4.55 (1.5)	0.391
SCr (μmol/L), mean (SD)	68.7 (22.8)	79.6 (26.5)	61.7 (16.8)	*< 0.001*
Urea (μmol/L), mean (SD)	5.1 (4.1)	6.6 (6)	4.2 (1.5)	*< 0.001*
eGFR (mL/min/1.73 m^2^), mean (SD)	89.9 (13.3)	68.39 (11.6)	100.3 (15.5)	*< 0.001*
ALT (U/L), mean (SD)	25.9 (24)	23.1 (12.9)	27.7 (29)	*0.003*
AST (U/L), mean (SD)	24.9 (30.5)	23.3 (13.3)	25.9 (37.5)	0.174
Albumin (g/L), mean (SD)	37.9 (4.4)	37.3 (3.9)	38.4 (4.6)	*< 0.001*
Vitamin D (mmol/L), mean (SD)	43.6 (24.6)	43.4 (24)	43.7 (25)	0.846
Calcium (mg/dL), mean (SD)	2.35 (1.2)	2.32 (0.16)	2.37 (1.5)	0.446
Sodium (mg/dL), mean (SD)	137.62 (6.1)	137.36 (7.3)	137.78 (5.2)	0.295
Potassium (mg/dL), mean (SD)	4.31 (0.4)	4.38 (0.5)	4.26 (0.3)	*< 0.001*

Significant values are in italics.

*BMI* body mass index, *SBP* systolic blood pressure, *DBP* diastolic blood pressure, *HbA1c* glycosylated hemoglobin A1C, *TC* total cholesterol, *TG* triglycerides, *LDL-C* low-density lipoprotein cholesterol, *HDL-C* high-density lipoprotein cholesterol, *SCr* serum creatinine, *eGFR* estimated glomerular filtration rate, *ALT* alanine transaminase, *AST* aspartate aminotransferase, *CKD* chronic kidney disease.

CKD: eGFR < 60 mL/min/1.73 m^2^ for ≥ 3 months.

No CKD: eGFR > 60 mL/min/1.73 m^2^ for ≥ 3 months.

*p values obtained from independent samples *t*-tests for continuous variables.

### Analyses of risk factors for and predictors of time to stage 3–5 CKD among diabetic patients

Table 6 presents the univariate and multivariate Cox regression analyses for the risk factors associated with the development of stage 3–5 CKD.

**Table 6:** Adjusted and unadjusted hazard ratios (HRs) and 95% confidence intervals (CIs) of predictors of developing stage 3–5 CKD.

Factors	Univariable (*N* = 1003)				Multivariable (*N* = 1003)			
	Unadjusted				Adjusted^b^			
	HR	95% CI		*p* value	HR	95% CI		*p* value
Age	1.003	1.001	1.004	< 0.001	1.003	1.001	1.006	0.031
Gender								
Female	1.00				1.00			
Male	1.212	0.991	1.482	0.062	0.815	0.643	1.033	0.091
Smoking	1.025	0.706	1.488	0.895	Not applicable^a^	–	–	–
Family history of DM	1.218	0.910	1.632	0.185	Not applicable^a^	–	–	–
Hypertension	5.067	3.116	8.241	< 0.001	1.665	1.015	2.731	0.043
Dyslipidemia	1.197	0.833	1.719	0.330	Not applicable^a^	–	–	–
Ischemic heart disease	2.681	2.123	3.387	< 0.001	1.539	1.194	1.984	0.001
BMI	0.986	0.971	1.002	0.190	Not applicable^a^	–	–	–
SBP	1.002	1.001	1.003	< 0.001	0.999	0.998	1.001	0.460
DBP	0.984	0.976	0.993	< 0.001	1.006	0.998	1.014	0.138
HbA1c	1.049	1.019	1.080	0.001	0.958	0.906	1.014	0.142
TC	0.981	0.914	1.053	0.602	Not applicable^a^	–	–	–
TG	1.021	0.969	1.077	0.432	Not applicable^a^	–	–	–
LDL-C	0.987	0.932	1.045	0.646	Not applicable^a^	–	–	–
HDL-C	0.787	0.584	1.060	0.114	Not applicable^a^	–	–	–
TC/HDL-C ratio	1.040	0.978	1.106	0.210	Not applicable^a^	–	–	–
SCr	1.027	1.024	1.031	< 0.001	1.008	1.003	1.013	0.001
Urea	1.047	1.038	1.057	< 0.001	1.002	0.982	1.022	0.840
eGFR	0.941	0.937	0.945	< 0.001	0.943	0.939	0.948	< 0.001
ALT	0.987	0.978	0.995	0.001	0.999	0.992	1.005	0.730
AST	0.996	0.988	1.004	0.281	Not applicable^a^	–	–	–
Albumin	0.964	0.946	0.982	< 0.001	0.985	0.959	1.012	0.278
Vitamin D	1.00	0.995	1.005	0.944	Not applicable^a^	–	–	–
Calcium	0.926	0.697	1.230	0.597	Not applicable^a^	–	–	–
Sodium	0.992	0.980	1.004	0.193	Not applicable^a^	–	–	–
Potassium	1.790	1.419	2.260	< 0.001	1.194	0.947	1.504	0.134
Diuretics	1.824	1.492	2.230	< 0.001	1.092	0.879	1.355	0.426
ACEIs	1.227	0.986	1.528	0.067	0.857	0.599	1.226	0.399
ARBs	1.400	1.144	1.713	0.001	0.848	0.605	1.187	0.337
Alpha-blockers	1.149	0.816	1.620	0.426	Not applicable^a^	–	–	–
Beta-blockers	2.511	2.054	3.069	< 0.001	1.375	1.105	1.710	0.004
Calcium channel blockers	1.800	1.468	2.207	< 0.001	1.113	0.892	1.389	0.342
Sulfonylureas	1.080	0.883	1.322	0.452	Not applicable^a^	–	–	–
Thiazolidinedione	1.196	0.929	1.542	0.165	Not applicable^a^	–	–	–
Dipeptidyl peptidase	1.032	0.844	1.262	0.757	Not applicable^a^	–	–	–
Biguanide	0.540	0.367	0.794	0.002	0.774	0.514	1.164	0.218
Alpha glucosidase	1.220	0.760	1.957	0.411	Not applicable^a^	–	–	–
Insulin	1.824	1.472	2.261	< 0.001	1.207	0.938	1.554	0.144
Statin								
No statin	1.00				1.00			
Low/moderate intensity statin	1.268	0.842	1.910	0.256	Not applicable^a^	–	–	–
High intensity statin	1.611	1.036	2.506	0.034	0.990	0.627	1.564	0.967

*DM* diabetes mellitus, *BMI* body mass index, *SBP* systolic blood pressure, *DBP* diastolic blood pressure, *HbA1c* glycosylated hemoglobin A1C, *TC* total cholesterol, *TG* triglycerides, *LDL-C* low-density lipoprotein cholesterol, *HDL-C* high-density lipoprotein cholesterol, *SCr* serum creatinine, *eGFR* estimated glomerular filtration rate, *ALT* alanine transaminase, *AST* aspartate aminotransferase, *ACEIs* angiotensin-converting enzyme inhibitors, *ARBs* angiotensin receptor blockers, *HR* hazard ratio, *CI* confidence interval.

^a^*p* value > 0.1 in the initial univariable analysis; predictor was not included in the multivariable analysis.

^b^Multivariable Cox regression model, adjusted for all predictors in the final model selected using backward selection.

The results of the univariate Cox regression models showed that older age; a history of hypertension; a history of ischemic heart disease; higher levels of SBP, HbA1c, SCr, urea, and potassium; lower levels of DBP, eGFR, ALT, and albumin; and the use of diuretics, ACEIs, ARBs, beta-blockers, calcium channel blockers, insulin, and high intensity statins increased the risk of developing stage 3–5 CKD. In contrast, a lower risk of stage 3–5 CKD was associated with the use of biguanides.

In the multivariate Cox prediction model, after backward stepwise selection, significant predictors of increased risk of stage 3–5 CKD included older age (hazard ratio (HR) 1.003, 95% confidence interval (CI) 1.001–1.006, *p* = 0.031), a history of hypertension (HR 1.66, 95% CI 1.015–2.73, *p* = 0.043), a history of ischemic heart disease (HR 1.54, 95% CI 1.19–1.98, *p* = 0.001), higher SCr levels (HR 1.008, 95% CI 1.003–1.013, *p* = 0.001), lower eGFR levels (HR 0.943, 95% CI 0.939–0.948, *p* < 0.001), and the use of beta-blockers (HR 1.37, 95% CI 1.11–1.71, *p* = 0.004).

Table 7 presents the results of multivariable Fine and Gray competing risk regression. Among the 39 variables we considered, the LASSO selection technique pinpointed 6 that emerged as predictors for the progression of stage 3–5 CKD in our multivariable competing risk regression model. The HRs, 95% confidence intervals (CIs), and *p* values associated with these variables are presented in Table 7.

**Table 7:** Multivariable Fine and Gray competing risk regression analysis model for developing stage 3–5 CKD.

Factors	Multivariate (*N* = 1003)			
	Adjusted			
	HR	95% CI		*p* value
Age	1.005	1.002	1.009	0.026
Hypertension	1.691	1.032	2.771	0.037
Ischemic heart disease	1.491	1.161	1.915	0.002
SCr	1.006	1.002	1.010	0.003
eGFR	0.943	0.938	0.947	< 0.001
Beta-blockers	1.389	1.117	1.727	0.003

The predictors were scaled using *z*-score transformation, and HRs should be interpreted as 1 SD change in the values of the parameter.

*HR* hazard ratio, *CI* confidence interval.

Several factors were found to increase the risk of developing stage 3–5 CKD: advancing age (HR 1.005, 95% CI 1.002–1.009, *p* = 0.026), a history of hypertension (HR 1.69, 95% CI 1.032–2.8, *p* = 0.037), a history of heart disease (HR 1.49, 95% CI 1.16–1.92, *p* = 0.002), elevated levels of SCr (HR 1.006, 95% CI 1.002–1.010, *p* = 0.003), decreased eGFR (HR 0.943, 95% CI 0.938–0.947, *p* < 0.001), and the use of beta-blockers (HR 139, 95% CI 112–173, *p* = 0.003).

The results of the Fine and Gray competing risk regression analysis were consistent with the findings of the standard multivariable Cox regression analysis.

## Discussion

To our knowledge, this is the first study to assess the risk factors for developing stage 3–5 CKD in patients with type 2 DM in the UAE. The outcomes of this research underscore the significance of baseline eGFR and SCr levels as important predictors of the onset of stage 3–5 CKD. Specifically, baseline eGFR has been identified as an important predictor of the development of these specific stages of the disease across both the general population and high-risk groups, inclusive of patients diagnosed with DM^40–44^. A reduction in eGFR below a critical threshold initiates a detrimental cycle, exacerbating renal impairment and contributing to hypertension, which subsequently accelerates the loss of nephrons^45^.

Our study also indicated that factors such as advancing age, a prior diagnosis of hypertension, and the presence of ischemic heart disease were independent risk factors for the development of stage 3–5 CKD. In a similar vein, various studies have identified age, smoking, obesity, dyslipidemia, and hypertension as independent risk factors for advancing to stage 3–5 CKD^40,46–53^. Notably, beyond the age of 50, the lifetime risk for the onset of CKD escalates by approximately 40%, with potential further increases when additional risk factors like obesity, high blood pressure, or DM^54–58^ are present. Furthermore, a substantial body of research has consistently affirmed a robust linkage between high blood pressure and CKD^49,50,59^. This underscores the need for comprehensive management of these risk factors within the context of DM care, aligning with findings from previous research^60–62^.

The utilization of beta-blockers in patients with chronic renal disease has been a topic of ongoing debate, primarily due to concerns about the potential to decrease cardiac output. Theoretically, a reduction in cardiac output could lead to diminished blood flow in the renal arteries, adversely affecting kidney perfusion^63^.

Interestingly, within our patient cohort, the use of beta-blockers was identified as a risk factor for advancing to stage 3–5 CKD. This statistical linkage might be attributed to the potential of beta-blockers to diminish cardiac output, subsequently leading to compromised renal perfusion—a condition posited to exert adverse effects on patients with CKD^64^. Furthermore, traditional beta-blockers such as propranolol, atenolol, and metoprolol have been documented to result in reduced eGFR and diminished renal blood flow. This reduction is a direct consequence of their capacity to lower cardiac output and increase peripheral vascular resistance, an effect intensified by the lack of a blockade at alpha-1 receptors^65^.

This particular finding may explain why beta-blockers were not extensively used in the current study (only 30% of the individuals in the study cohort were prescribed beta-blockers). This limited prescription rate could be attributed to the tolerability of the agents^66^. This trend aligns with other research, which has noted that only 20–30% of patients diagnosed with CKD are prescribed beta-blockers^67,68^.

Despite the concerns and limited prescription rates associated with beta-blockers in the context of CKD, it is important to note that cardiovascular disease is a predominant cause of death among CKD patients. Beta-blockers have been shown to reduce mortality rates in patients who have experienced a myocardial infarction as well as in those with chronic systolic heart failure^63,69,70^. Considering the elevated incidence of coronary artery disease and heart failure within the CKD patient demographic, beta-blockers could potentially offer substantial benefits to this patient population. This assertion is backed by a comprehensive systematic review of eight clinical studies, which concluded that, in CKD patients with heart failure, beta-blockers significantly reduced both all-cause mortality (relative risk (RR) 0.72, CI 0.64–0.80) and cardiovascular mortality (RR 0.66, CI 0.49–0.89) compared to placebo^69^. This intricate balance between renal and cardiovascular outcomes necessitates careful consideration by clinicians, who are tasked with developing personalized treatment plans that weigh the renal implications against the cardiovascular advantages^53,56^.

In the realm of clinical practice, the challenge of making early predictions regarding stage 3–5 CKD based on eGFR values is substantial^71^. Addressing this issue provides healthcare practitioners with a valuable tool to identify patients at risk, enabling the swift implementation of lifestyle modifications, adjustments in medication, and the initiation of preventative strategies aimed at preserving renal function^72^. Engaging in such preemptive actions has the potential to improve patient care, promote favorable outcomes, and alleviate the overall strain on healthcare systems^73^. Our study underscores the challenges of accurately predicting stage 3–5 CKD using eGFR values in clinical settings. There is a critical need for more precise predictive tools to facilitate early and effective interventions. Advancing our understanding of the combined effects of various risk factors on CKD progression is essential for improving risk prediction models, thereby offering the potential to transform clinical practice by enabling the early identification and preventative management of high-risk patients.

The main strength of our study is its significant contribution to the existing literature on CKD and its particular focus on the population of the UAE. By shedding light on epidemiological patterns and identifying specific risk factors within this demographic, our research provides invaluable insights for healthcare providers in the region. This tailored approach, informed by population-specific data, is crucial for designing interventions that are aligned with the unique health profile and healthcare framework of the UAE.

Another notable strength of our research is its distinction as the first longitudinal study to assess the epidemiology of stage 3–5 CKD, alongside specific risk factors, in a population of type 2 DM patients who are nationals of the UAE. This study is further characterized by a considerable follow-up duration of 11 years. Ideally, an extended follow-up period would be advantageous, as it would facilitate a more comprehensive understanding of the long-term risk factors associated with the progression to stage 3–5 CKD.

Another merit of the research is the methodology employed for diagnosing stage 3–5 CKD, which was based on two consecutive eGFR readings < 60 mL/min/1.73 m^2^, spaced ≥ 3 months apart. This approach could help account for the intra-individual variability associated with eGFR, leading to a more precise representation of renal function. In addition, the CKD-EPI equation was utilized to define the study’s outcome, a method deemed more accurate than the Modification of Diet in Renal Disease Study equation, as corroborated by the majority of existing studies^74–77^. Moreover, this study utilized documented anthropometric and laboratory measurements, as opposed to relying on self-reported data, for both the predictor variables and the outcomes, thereby ensuring a higher level of data reliability.

### Study Limitations

This investigation is subject to several limitations. First, the retrospective nature of the study could have introduced biases and limitations in data quality, which might have been mitigated through a prospective study design involving standardized measurements of laboratory variables and anthropometric parameters.

Second, the study did not explore certain risk factors, such as albuminuria, despite numerous studies highlighting its predictive value for the development of kidney failure^50,78–81^. In the UAE, non-nephrologist physicians have reported that albuminuria is not routinely measured in clinical practice, with nearly 80% relying solely on eGFR as a screening tool for CKD^82^.

Third, our study’s sample size was relatively modest compared to other investigations in this field. Fourth, the study’s findings were derived from data collected at a single hospital, which may not be representative of the broader international context. Fifth, given the study’s reliance on a retrospective review of patient records, it is possible that not all factors influencing survival probabilities were captured.

## Conclusion

The current study emphasizes the necessity for a holistic strategy in the prevention, identification, and management of CKD. Healthcare professionals and policymakers should consider the complex and multifaceted nature of this disease, as well as the interrelationships between various risk factors. Implementing preventative measures, initiating early interventions, and developing personalized care plans tailored to address specific risk factors are imperative for reducing the impact of CKD. Additionally, the unforeseen findings related to eGFR highlight the ongoing need for research to deepen our understanding of the complexities of kidney disease. This study provides valuable insights into the factors that contribute to CKD risk, laying a solid foundation for future research aimed at improving strategies for the prevention and management of kidney disease.

### Supplementary Information

Supplementary Information.

## Data Availability

The original contributions presented in the study are included in the manuscript, and further inquiries regarding the data can be directed to the corresponding authors.
