# Supplementary material for: Predictors of chronic kidney disease survival in type 2 diabetes: a 12-year retrospective cohort study utilizing estimated glomerular filtration rate
Source: Sci Rep. 2024 Apr 19;14:9014. doi: 10.1038/s41598-024-58574-x (PMC11031608; doi:10.1038/s41598-024-58574-x)
Supplement: Supplementary file 1 — Supplementary Information. [file 41598_2024_58574_MOESM1_ESM.docx]

**Supplementary Materials**

## 2.6 Research tool

A data collection form (in excel sheet) was designed for the data collection purposes. The data collected for each case subject included subject ID number and baseline covariates of the patients. The patients’ characteristics was collected at baseline. The collected data included: (i) Socio-demographics, (ii) detailed medical history, (iii) anthropometric measurements, (iv) laboratory analyses and clinical parameters, (v) disease characteristics and medications.

## 2.7 Operational definition

- **Baseline characteristics**: Age, gender, smoking status.
- **Clinical parameters:** HbA1c%, SBP (mmHg), DBP (mmHg), BMI, LDL-C (mmol/L), (kg/m^2^), TC/ HDL-C Ratio, Triglyceride (mmol/L), TC (mmol/L), TG (mmol/L), SCr (μmol/l), Urea (μmol/l), eGFR (mL/min/1.73m2), ALT (U/L), AST (U/L), Albumin (g/L), Vitamin D (mmol/L), Calcium (mg/dL), Sodium (mg/dL) and Potassium (mg/dL).
- **Disease Characteristics**: Duration of DM, presence of chronic kidney disease, hypertension, family history of DM, Dyslipidemia and Ischemic heart disease.
- **Treatment/concurrent medications**: Use of insulin, sulfonylurea, metformin, Thiazolidinediones (TZD), Dipeptidyl Peptidase, Alpha glucosidase, statin, use of anti-hypertensive drugs such as Diuretics, ACEIs, ARBs, Alpha-blockers, Beta-blockers, Calcium channel blocker and no drug therapy.
- **Diagnosis of the risk of cardiovascular disease (CVD):** Patients were considered as having CVD if they had a diagnosis of coronary heart disease (angina, prior myocardial infarction, angioplasty of the coronary arteries or coronary artery surgery), cerebrovascular accidents or peripheral vascular disease. Diagnosis established by a physician.
- **Diagnosis of diabetes mellitus (DM):** American Diabetes Association criteria of HbA1c ≥ 6.5%will be used to define DM. Diagnosis established by a physician.
- **Hypertension (HTN):** defined as systolic blood pressure ≥ 140 mm Hg, diastolic blood pressure ≥ 90 mm Hg or the use of antihypertensive medications. Diagnosis established by a physician.
- **Dyslipidemia** is defined as the presence of one or more of the following: triglyceride ≥ 1.69 mmol/L, total cholesterol ≥ 5.17 mmol/L, low-density lipoprotein cholesterol ≥ 3.36 mmol/L, high-density lipoprotein cholesterol < 1.03 mmol/L or documented treatment with lipid-lowering medications. Diagnosis established by a physician.
- **Body mass index (BMI):** calculated as weight (kg) divided by height (m) squared and obesity is defined as BMI ≥ 30 kg/m^2^.
- **Smoking history** is positive if there was a current or history of smoking of tobacco.
- **CKD stages 3-5:** were defined by using the KDIGO 2012 Clinical Practice Guideline for the Evaluation and Management of Chronic Kidney Disease was used in the study as an eGFR < 60 mL/min/1.73 m2 for ≥ 3 months
